# Hemiplegic migraine: genetics and pathophysiology

**DOI:** 10.1172/JCI208001

**Published:** 2026-09-15

**Authors:** Daniela Pietrobon

**Affiliations:** Department of Biomedical Sciences and Padova Neuroscience Center, University of Padova, Padova, Italy.

## Abstract

Rare monogenic subtypes of migraine with aura, which include an autosomal dominant form of hemiplegic migraine (HM), are caused by exonic mutations whose functional consequences can be studied in cellular and animal models of the disease. This allows investigation of the neurobiological mechanisms at the molecular, cellular, and circuit level. Here, I review current knowledge of the genetics and pathophysiology of HM. After considering the genes whose mutations cause familial HM (FHM) and discussing how the encoded proteins are affected by the mutations, I consider the mouse models generated by introducing human FHM mutations in the orthologous genes, *Cana1a*, *Atp1a2*, and *Scna1a*. I discuss their phenotypes, highlighting their shared increased susceptibility to experimentally induced cortical spreading depression (CSD, the phenomenon which underlies migraine aura and may trigger the headache mechanisms) and migraine-relevant pain behaviors. I examine the alterations in the cerebral cortex and the mechanisms underlying the facilitation of CSD in the mouse models as well as the alterations in the trigeminovascular pain pathway and their possible contributions to migraine-relevant pain phenotypes. Finally, I discuss the translational implications of the pathogenic mechanisms of CSD facilitation.

## Introduction

Migraine is a common disabling neurological disorder characterized by recurrent attacks of typically throbbing and unilateral, often severe, headache with associated features such as nausea and/or photophobia and phonophobia. In approximately one-third of patients, these symptoms are preceded by transient visual, sensory, or other CNS symptoms (migraine aura). When the aura includes motor weakness, the disorder is classified as hemiplegic migraine (HM) (1) (Table 1). Migraine is characterized by a global dysfunction in multisensory processing, which points to alterations in multiple brain networks in addition to those related to pain (2–4).

The mechanisms underlying migraine headache and aura are reasonably well understood and have been reviewed in detail elsewhere (2–9). Briefly, the headache depends on activation and sensitization of the trigeminovascular system, with a pivotal role for calcitonin gene related peptide (CGRP) release. The neurophysiological correlate of migraine aura is a slowly propagating (2–5 mm/min), self-sustaining wave of nearly complete depolarization of brain cells, named cortical spreading depression or spreading depolarization (CSD). Accumulating evidence indicates that CSD can initiate the headache mechanisms, as it can activate and sensitize trigeminal nociceptors and the trigeminovascular pain pathway, produce dural and cortical inflammation, and induce trigeminal pain behavior in rodents that responds to migraine therapies (6, 9–11). However, although most migraine attacks originate in the brain, the neurobiological mechanisms underlying their onset and the susceptibility to CSD remain largely unknown.

Migraine is a complex multifactorial genetic disorder, brought about by a combination of multiple genetic variants, each with a small effect size, and internal and external environmental factors (12, 13). Rare monogenic subtypes of migraine with aura (MA), which include familial HM (FHM), are caused by single exonic mutations (12, 13). Studies of the functional consequences of FHM mutations in cellular and animal models of the disease have enabled insights into the causative mechanisms at the molecular, cellular, and circuit level. Here, I review current knowledge of the genetics and pathophysiology of HM and discuss the translational implications.

## HM

HM is characterized by unilateral (rarely bilateral) transient motor weakness associated with at least another aura symptom (1) (Table 1). HM can occur sporadically (SHM) or as a familial (FHM) condition if at least one first- or second-degree relative has the same form of migraine. Clinical features of attacks are similar in SHM and FHM (14, 15). Headache is almost always present during attacks and it is often severe. Aura symptoms progress over 20–30 min and occur in succession, usually changing from visual symptoms to sensory, motor, aphasic, and finally basilar disturbances. Attacks typically begin during youth and their mean frequency is low (three attacks per year) but highly variable. Pure HM resembles MA except for the obligatory motor aura, increased frequency of other aura symptoms and the usually longer duration of aura and headache. Pure HM and MA attacks may alternate in patients and co-occur within families. Some patients show severe HM attacks with disturbances of consciousness (ranging from confusion and somnolence to profound coma) frequently associated with motor deficit and fever and sometimes with seizures and reversible brain edema (14, 15). Motor symptoms generally last less than 72 hours, but in severe attacks, hemiplegia and altered consciousness may persist for weeks. Some patients with HM have permanent neurological manifestations ranging from cerebellar signs (including gaze-evoked nystagmus, progressive ataxia and cerebellar atrophy), to early-onset epilepsy with partial or generalized seizures independent of HM attacks, to cognitive impairment (14, 15).

## HM genetics

A subset of patients with HM carry single pathogenic mutations in the *CACNA1A* (FHM1/SHM1), *ATP1A2* (FHM2/SHM2), or *SCN1A* (FHM3/SHM3) genes (Table 1) (16–18). SHM can be caused by de novo mutations in genes causing FHM or by inheritance of a mutation from an asymptomatic parent with FHM (19, 20). In sporadic cases, early-onset and presence of associated neurological symptoms are associated with increased likelihood of an FHM mutation (14, 19, 21). Although HM attacks are similar regardless of the gene involved, the causative gene may influence the spectrum of associated manifestations. For example, both febrile comas and cerebellar ataxia are more frequent in FHM1 than FHM2 and are not described in FHM3; in contrast to FHM1 and FHM2, HM attacks and seizures do not overlap in FHM3, in which elicited repetitive daily blindness (ERDB) is a type-specific comorbidity (14, 15, 22–24). Distinct mutations in the same gene can result in pure FHM/SHM with typical attacks or severe FHM/SHM with atypical attacks and chronic symptoms (25). However, family members with the same mutation can show wide variability in clinical presentation, suggesting that unknown genetic or environmental factors can influence phenotype (26).

Population-based studies of FHM/SHM and next-generation sequencing approaches in large cohorts of patients suspected of having HM show that only a minority of families/patients have pathogenic variants in *CACNA1A*, *ATP1A2*, or *SCNA1A* (27–31) (Table 2). In contrast, in a cohort of mainly Dutch families, causative mutations in the three FHM genes were present in 63% of symptomatic individuals (21). Patients without such mutation had a milder phenotype, more similar to nonhemiplegic MA, and were older at disease onset than patients carrying a causative mutation (21). In the Finnish population, the clinical symptoms of patients with HM (most of whom did not carry mutations in *CACNA1A*, *ATP1A2*, or *SCNA1A*) could not clearly distinguish them from patients with MA but rather reflected an extreme phenotype in the MA continuum (29).

Based on the relatively high frequency of likely pathogenic *PRRT2* gene variants in a large cohort of HM probands, Riant et al. (31) concluded that *PRRT2* should be regarded as the fourth autosomal dominant gene for FHM/SHM (22). Loss-of-function mutations in *PRRT2* are the leading cause for a wide spectrum of paroxysmal diseases, including benign familial infantile epilepsy (BFIE), infantile convulsions and choreoathetosis (ICCA), and paroxysmal kinesigenic dyskinesia (PKD) (32). The most common *PRRT2* variant in patients with HM is the truncating c.649dupC frameshift variation, which is the main causative mutation in PKD and BFIE and can also cause ICCA (31, 32). The vast majority of individuals with this loss-of-function mutation do not have HM (32). Thus, a *PRRT2* mutation alone does not seem sufficient to cause HM (21, 33), but since the frequency of HM in *PRRT2* mutation carriers appears too high to be incidental (32, 34), *PRRT2* might act as a main disease modifier gene for HM within a complex polygenic mechanism (21, 33). Indeed, an increased burden of common and/or rare variants in several genes contributes to familial aggregation or disease risk in large cohorts of families and patients with FHM referred for HM diagnosis without a disease-causing mutation in *CACNA1A*, *ATP1A2*, or *SCNA1A* (35–38). In a small number of patients with HM, mutations in other genes (mainly ion or solute carriers, including *SLC1A3* and *SLC4A4*) have been reported (13, 30, 39–42), but additional data are needed before they can be considered causal (12, 43). Recently, a heterozygous missense mutation in *SCN2A* was found to cosegregate with FHM in a four-generation pedigree, and additional *SCN2A* variants were found in a second family and a sporadic case (44). Overall, the findings suggest that FHM is a dominantly inherited monogenic disease in some patients but may be polygenic or oligogenic in others (13, 21, 29, 35–37).

## HM genes and proteins

### FHM1/SHM1.

*CACNA1A* encodes the pore-forming subunit of the voltage-gated calcium channel Ca_V_2.1 (or P/Q-type). This channel is widely expressed in the nervous system, where it plays a prominent role in initiating neurotransmitter release, particularly at central synapses (ref. 45 and references therein). Expression is particularly high in the cerebellum, which may explain the cerebellar symptoms in several patients with FHM1 (45).

**Figure 1:**
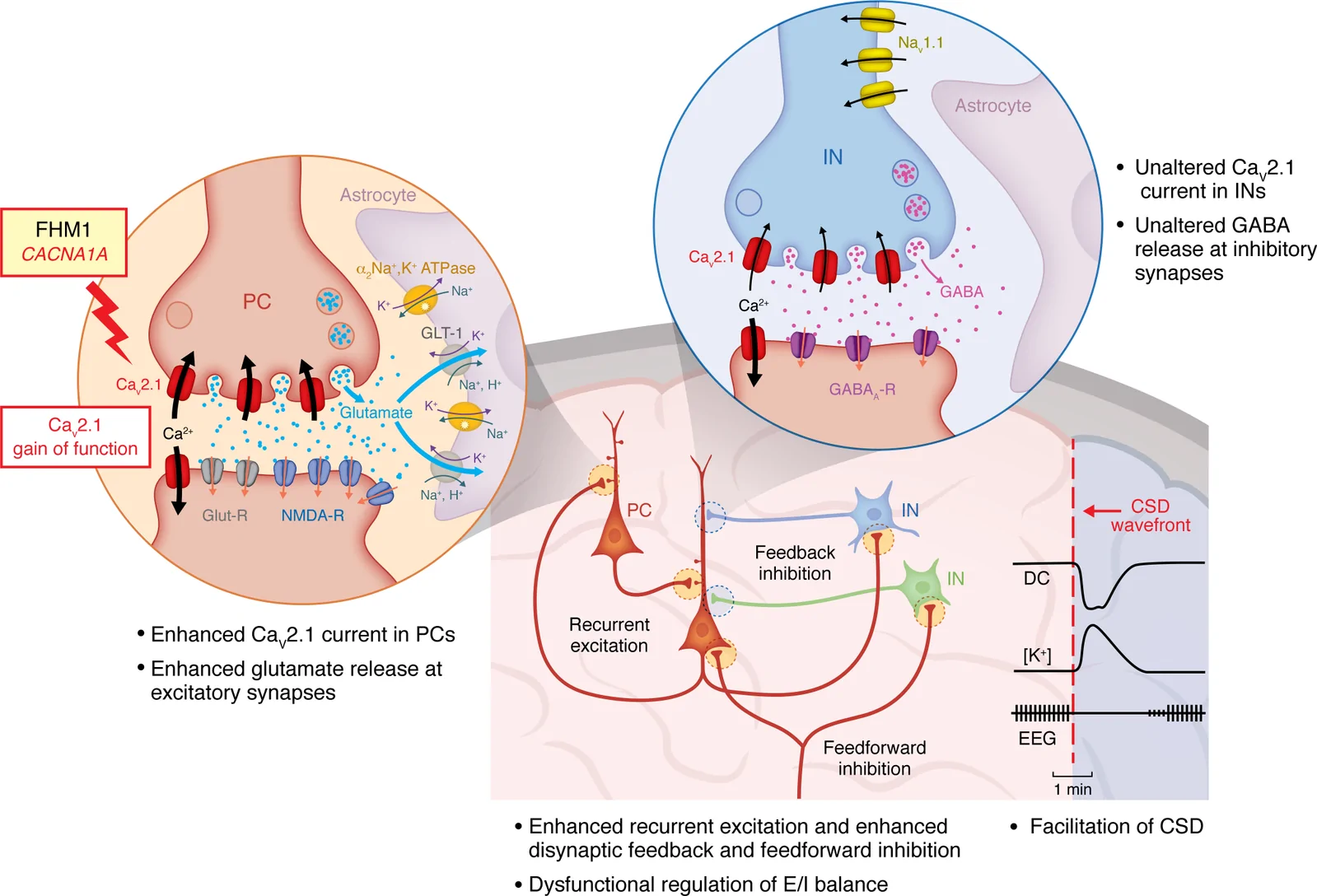
Functional alterations in the cerebral cortex in FHM1 knockin mouse models. In FHM1 mice, the Ca_V_2.1 current is increased in pyramidal cells, but unaltered in inhibitory interneurons. Excitatory synaptic transmission is increased due to enhanced probability of glutamate release, while inhibitory synaptic transmission is unaltered. Both recurrent excitation and disynaptic feedback and feedforward inhibition are enhanced, and the excitation/inhibition (E/I) balance may be shifted toward inhibition in certain conditions. The heightened Ca_V_2.1-dependent glutamate release may explain the enhanced susceptibility to experimentally induced CSD. Glut-R, glutamate receptor; NMDA-R, NMDA receptor; PC, pyramidal cell; IN, interneuron.

More than 40 missense mutations associated with FHM1/SHM1, affecting conserved amino acids in Ca_V_2.1, have been identified (46). FHM1/SHM1-associated variants are gain-of-function mutations, resulting in increased open probability and activation at lower voltages of human recombinant Ca_V_2.1 channels (45, 47, 48). Reduced G protein–mediated inhibition of mutant channels might lead to a further increase in Ca^2+^ influx during neuromodulation (49–51). The gain-of-function effect may depend on the specific Ca_V_2.1 splice variant and/or auxiliary subunit (52–54), and thus may be neuron-type specific. Indeed, in *Cacna1a*-knockin mice carrying FHM1 mutations, the Ca_V_2.1 current was enhanced in specific neurons, including cortical pyramidal cells and distinct trigeminal ganglion (TG) neurons (55–60), but was unaltered in others, including cortical inhibitory interneurons (58, 61) (Figure 1).

### FHM2/SHM2.

*ATP1A2* encodes the α_2_ Na^+^/K^+^ ATPase (NKA). This NKA isoform is expressed primarily in neurons during embryonic development and at the time of birth and almost exclusively in astrocytes in the adult brain, where it plays a key role in K^+^ and glutamate clearance during neuronal activity (ref. 62 and references therein). The fundamental role of α_2_ NKA in clearance of synaptically released glutamate relies on its colocalization and physical coupling with the glutamate transporters GLT-1 and GLAST at the astrocytic processes surrounding cortical excitatory synapses and its relatively low affinity for [Na+]^in^ (62–65). α_2_ NKA is also important for intracellular Na^+^ and Ca^2+^ homeostasis/signaling, pH regulation, and neuron-astrocyte neurometabolic coupling (66, 67).

**Figure 2:**
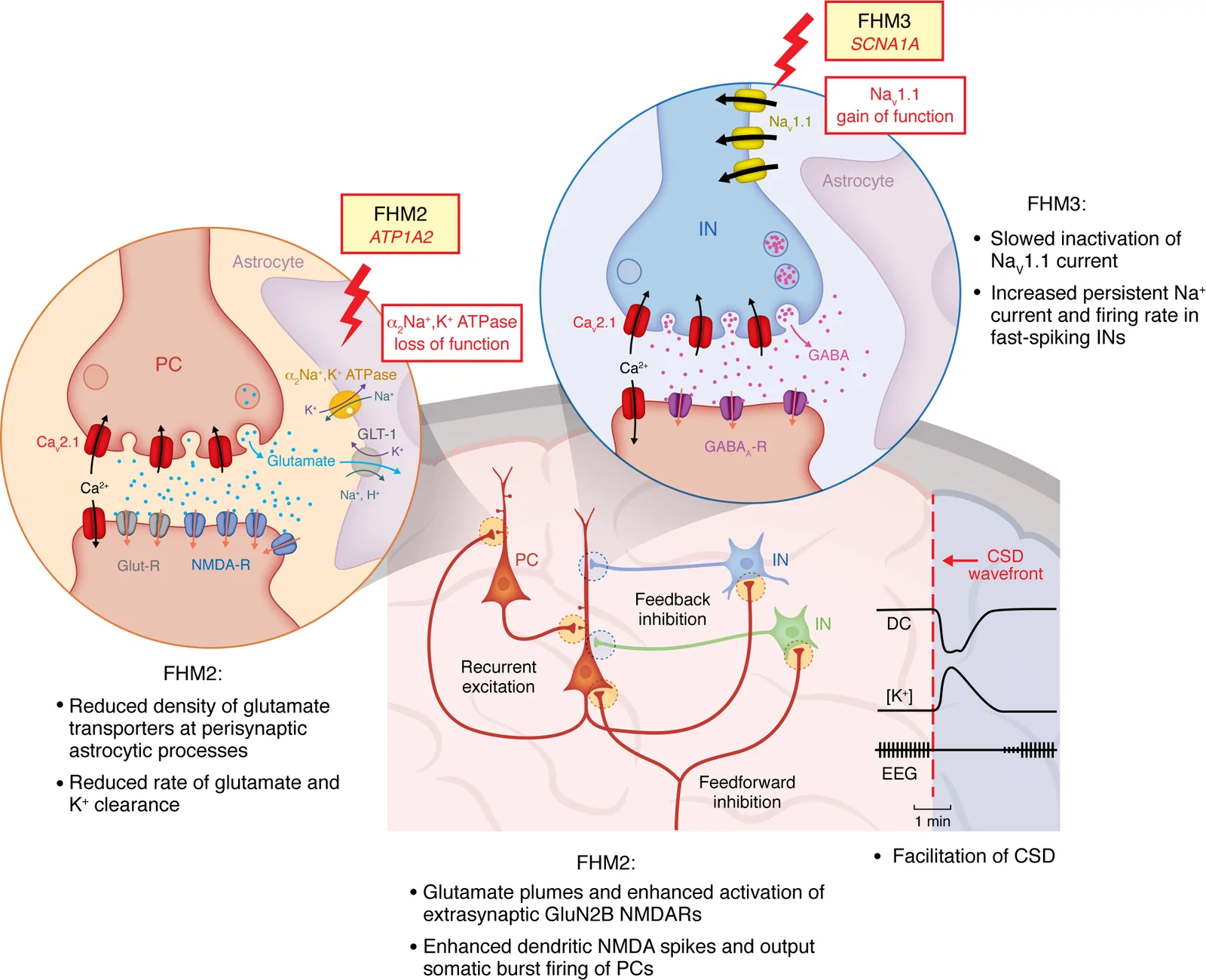
Functional alterations in the cerebral cortex in FHM2 and FHM3 knockin mouse models. In FHM2 mice, the 50% reduction of α_2_ NKA expression results in a similarly reduced density of glutamate transporters at perisynaptic astrocytic processes and in a reduced rate of clearance of both K^+^ and glutamate during neuronal activity. Spontaneous focal glutamate transients (glutamate plumes) occur in layer 1, as markers of inefficient glutamate clearance. The activation of extrasynaptic GluN1-N2B NMDARs in pyramidal cells elicited by stimulation of layer 1 afferents is enhanced and the generation of dendritic NMDA spikes in pyramidal cells is facilitated, leading to enhanced somatic burst firing of the pyramidal cells. The reduced rate of glutamate clearance and the increased activation of extrasynaptic GluN1-N2B NMDARs may explain the enhanced susceptibility to experimentally induced CSD. In FHM3 mice, inactivation of the Na_V_1.1 current is slowed, and the persistent Na^+^ current as well as the firing rate are increased in fast-spiking inhibitory interneurons. Glut-R, glutamate receptor; NMDA-R, NMDA receptor; PC, pyramidal cell; IN, interneuron.

More than 90 missense mutations in *ATP1A2* have been associated with FHM2/SHM2 (46), and these cause complete or partial loss-of-function of human recombinant α_2_ NKA (68–71) (Figure 2). The brain expression of α_2_ NKA in heterozygous knockin mice carrying FHM2 mutations was either 50% reduced or unaltered, depending on whether the mutations impaired membrane targeting and caused complete loss of function of recombinant pumps (W887R, G301R) or caused partial loss of function (T345) (72–77).

### FHM3/SHM3.

*SCNA1A* encodes the pore-forming subunit of the voltage-gated sodium channel Na_V_1.1. This neuronal channel is predominantly expressed in inhibitory interneurons in several brain areas; it predominantly localizes at the axon initial segment where it plays a key role in interneuron excitability, particularly in sustaining high-frequency firing (78–81). Of potential relevance for migraine pain, Na_V_1.1 is also expressed in (and regulates excitability of) sensory mechanoreceptive A-δ fibers and a subset of TG neurons (82).

More than 10 missense mutations in *SCNA1A* have been associated with FHM3/SHM3 (46, 83). The initial functional studies of FHM3 mutations on human recombinant Na_V_1.1 channels generated conflicting results (reviewed in ref. 24), but it is now understood that FHM3 mutations produce an overall gain-of-function effect, mainly due to destabilization of the Na_V_1.1-inactivated state (24, 84–87). Accordingly, knockin of a FHM3 mutation in mice resulted in increased persistent Na^+^ current and slower Na^+^ current inactivation in GABAergic neurons (88) (Figure 2). Stabilization of the Na_V_1.1-inactivated state by the late Na^+^ current inhibitor GS967 rescued the gain of function of recombinant human Na_V_1.1 channels (86, 87), and GS967 inhibited the persistent Na^+^ current in fast-spiking interneurons of FHM3 mice (88).

## Genetic mouse models of FHM

Eight FHM mouse models have been generated by introducing human mutations in the orthologous genes (Table 3) (55, 56, 72, 73, 77, 88–90). Most carry mutations causing pure HM, and heterozygous mice of these lines did not show overt behavioral phenotypes. Homozygous *Cacna1a^R192Q^* mice also did not display an overt phenotype (55), but homozygous *Scna1a^L1649Q^* and *Atp1a2^T345A^* mice had reduced lifespan (77, 88), and homozygous *Atp1a2^W887R^* mice (with barely observable brain expression of α_2_ NKA) died at birth (72). Heterozygous *Scna1a*^L263V^ mice (90), which carry a mutation identified in two families in which most carriers had pure HM and one family branch showed independent attacks of epilepsy (91), died prematurely due to spontaneous brainstem depolarization-associated apnea, which was prevented by administration of GS967 (87, 90). Notably, GS967-sensitive life-threatening apneic events were reported in an infant homozygous for *SCNA1A*^L263V^, whereas the heterozygous family members had pure FHM (87).

*Cacna1a^S218L^* and *Atp1a2^G301R^* (56, 73) mice carry mutations associated with severe HM attacks, which include prolonged coma/torpor, confusional state, cerebral edema (often triggered by minor head trauma in S218L carriers), fever, seizures, and transient or permanent cerebellar signs, including progressive ataxia (76, 92–94). Homozygous *Cacna1a^S218L^* mice model the main features of the severe human clinical syndrome, including spontaneous attacks of hemiparesis and sometimes fatal seizures, brain edema after mild head impact, and ataxia (56, 95). The severe clinical/behavioral phenotype correlates with the particularly low activation threshold of human S218L Ca_V_2.1 channels (48). Heterozygous *Atp1a2^G301R^* mice show several behavioral alterations, including increased startle response to aversive acoustic stimuli, decreased sociability, stress-induced depression-like phenotypes, and increased compulsive behavior specifically in females (73). *Atp1a2^G301R^* mice also exhibit dysfunction of the glymphatic system, possibly linked to excessive neurovascular coupling (96, 97).

### Increased susceptibility to CSD.

A key migraine-relevant phenotype shared by all FHM mouse models is increased susceptibility to CSD induced by focal stimulation (although only as a trend in heterozygous *Atp1a2^T345A^* and *Atp1a2^E700K^* mice) (Table 3) (55–57, 64, 72, 77, 88–90, 98–102). The rate of CSD propagation was increased in all FHM1 and FHM2 models. In *Cacna1a^R192Q^* and *Cacna1a^S218L^* mice, a single CSD caused contralateral hemiplegia with leaning and circling, and CSD readily propagated into the striatum, suggesting facilitation of corticostriatal CSD propagation as a possible explanation for the motor deficits (99, 103, 104). The typical reduction of cerebral blood flow after CSD was more prolonged and the metabolic oxygen consumption during CSD was larger and resulted in a larger decrease of tissue oxygenation in *Cacna1a^R192Q^* mice compared with WT mice (105). In both FHM1 models, CSD facilitation was larger in female than male mice (101), and both stress hormone administration and relief from stress (but not acute or chronic stress) facilitated CSD (106, 107). These mice also showed increased susceptibility to CSDs after traumatic brain injury (108).

Correlating with the clinical phenotypes, facilitation of CSD initiation and propagation as well as severity of the post-CSD neurological motor deficits and propensity of CSD to propagate into subcortical structures were greater in *Cacna1a^S218L^* mice than in *Cacna1a^R192Q^* mice (56, 101, 103, 104, 108). Moreover, multiple CSDs after a single CSD-inducing stimulus were frequently observed in *Cacna1a^S218L^* (but not *Cacna1a^R192Q^*) mice, and such events were more frequent in homozygotes than in heterozygotes (56, 99). Homozygous *Cacna1a^S218L^* and heterozygous *Atp1a2^G301R^* mice also developed generalized seizures after CSDs (98, 101). These unique CSD features might contribute to the severe clinical phenotype in humans carrying S218L or G301R mutations.

Long-term recordings with implanted electrodes revealed only rare spontaneous CSDs in homozygous *Ccacna1a^R192Q^* and both heterozygous *Cacna1a^S218L^* and *Atp1a2^T345A^* mice, whereas frequent spontaneous CSDs, typically originating from the hippocampus and inhibited by GS967, were revealed in homozygous *Atp1a2^T345A^* mice (77, 99). Frequent spontaneous CSDs were also recorded in heterozygous *Scna1a^L263V^* mice (90).

The mechanisms underlying the enhanced susceptibility to CSDs in the FHM1 and FHM2 models involve alterations in glutamate signaling. In the FHM1 mice, excitatory synaptic transmission at different intracortical and thalamocortical synapses was enhanced, due to increased probability of glutamate release (57, 109–111). In contrast, GABAergic transmission at different cortical inhibitory synapses, although initiated by Ca_V_2.1 channels, was unaltered (57, 61, 109, 111) (Figure 1). A causative link between gain of function of glutamate release at cortical synapses and facilitation of CSD was established in *Cacna1a^R192Q^* mice (57). In addition to a larger evoked glutamate release in *Cacna1a^S218L^* mice compared with *Cacna1a^R192Q^* mice, a larger metabolic burden on cortical neurons likely contributes to the larger facilitation of CSD and the unique CSD features in *Cacna1a^S218L^* mice (109). Indeed, in contrast with R192Q, the S218L mutation leads to Ca_V_2.1 channel opening at resting potentials and to very slow channel inactivation during prolonged depolarizations (48, 59, 109, 112). Opening of S218L Ca_V_2.1 channels at resting potentials also contributes to the irregular spontaneous Purkinje cell firing and the ataxia phenotype in *Cacna1a^S218L^* mice (113).

The importance of excessive glutamatergic transmission in CSD susceptibility is underscored and supported by the functional alterations in the cerebral cortex of *Atp1a2^W887R^* mice. In this model, the 50% reduction of α_2_ NKA expression results in a similarly reduced density of GLT-1 glutamate transporters at cortical perisynaptic astrocytic processes and in a reduction of the rate of clearance of both K^+^ and glutamate at cortical synapses during neuronal activity (64, 102, 114, 115). As a consequence, awake *Atp1a2^W887R^* mice showed spontaneous high-amplitude, focal glutamate transients (“glutamate plumes”) not present in WT mice (102). The activation of NMDA receptors (NMDARs) in layer 2/3 pyramidal cells elicited by stimulation of layer 1 afferents was increased and prolonged in *Atp1a2^W887R^* mice, owing to activation of extrasynaptic diheteromeric GluN1-N2B receptors (115). In addition, the generation of long-lasting NMDA spikes in the tuft dendrites of L5 pyramidal cells was facilitated, leading to enhanced output somatic burst firing (114) (Figure 2).

The reduced rate of glutamate clearance at cortical synapses can account for most of the facilitation of CSD initiation and a large fraction of the facilitation of CSD propagation in *Atp1a2^W887R^* mice (64). The remainder is likely related to the reduced rate of K^+^ clearance in this model. Extracellular glutamate imaging at the site of CSD initiation in awake head-fixed mice revealed that an increase in basal glutamate and in frequency of glutamate plumes precedes and may predict CSD initiation (102). The critical threshold level of glutamate at which CSD ignited was similar in *Atp1a2^W887R^* and WT mice but was reached with a lower stimulation intensity and more rapidly in *Atp1a2^W887R^* mice, accounting for their facilitated CSD initiation (102).

Recent studies showed that a critical threshold level of Ca_V_2.1-dependent NMDAR activation is necessary for CSD ignition; this critical level is similar in *Cacna1a^R192Q^* and WT mice but is reached with a lower stimulation intensity and more rapidly in *Cacna1a^R192Q^* mice (116, 117). These findings point to excessive activation of NMDARs by low-intensity stimulation, as the key mechanism underlying facilitation of CSD initiation in FHM1 and FHM2. Indeed, both the increased and prolonged activation of NMDARs and the facilitation of CSD initiation in A*tp1a2*^W887R^ mice were rescued by specific inhibition of GluN1-N2B NMDARs (115).

**Figure 3:**
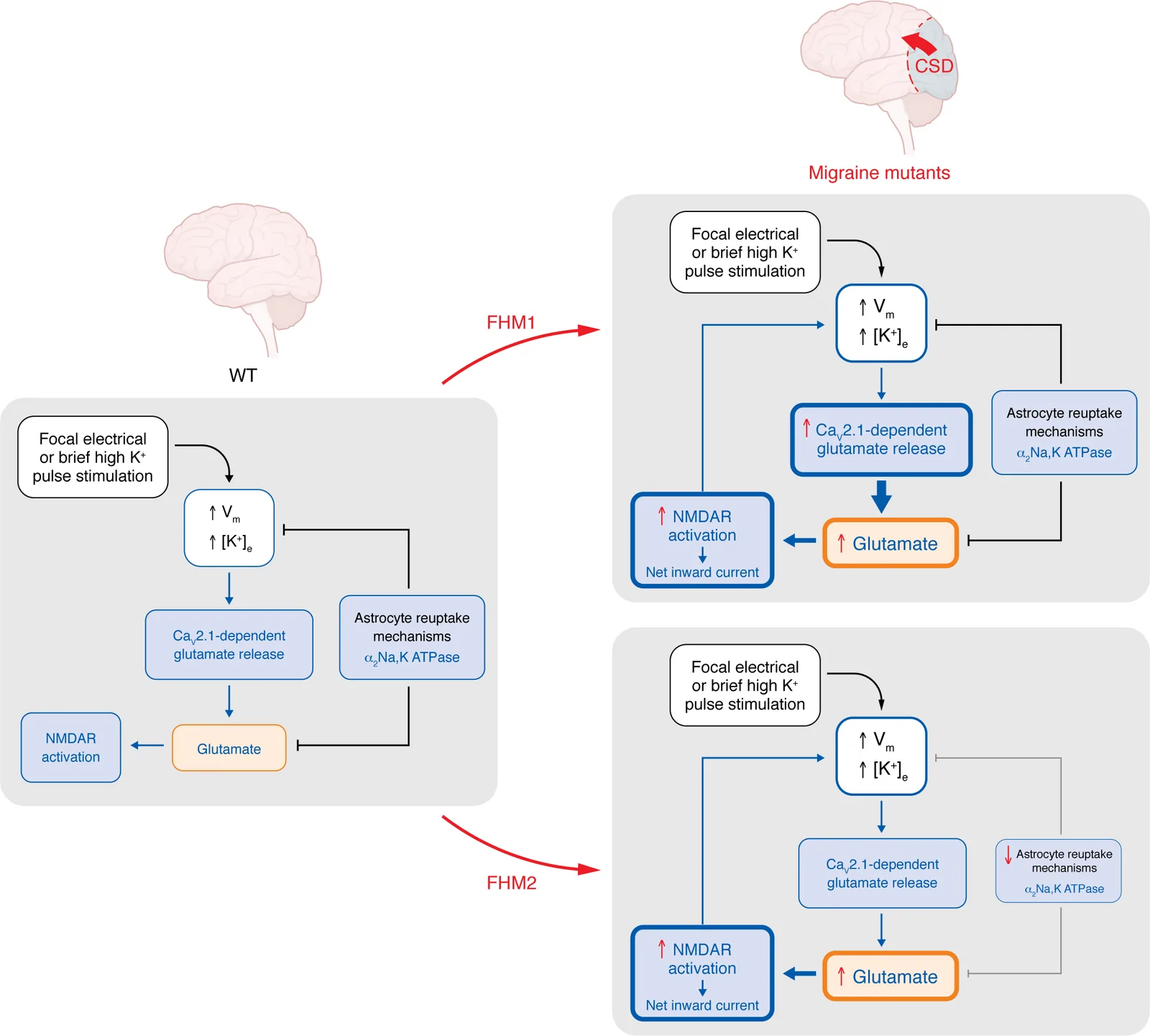
Excessive glutamatergic neurotransmission and excessive activation of NMDA receptors underlie the facilitation of CSD in FHM1 and FHM2 mouse models. The threshold depolarizing stimulus that initiates CSD in FHM1 and FHM2 mice does not induce CSD in WT mice. In the mutants, this stimulus increases glutamate and activates NMDARs above the critical levels necessary to generate a net self-sustaining inward current and initiate the CSD positive feedback cycle. The amplificatory cycle of glutamate/K^+^ release is potentiated as a consequence of either increased glutamate release (FHM1 models) or reduced glutamate clearance (FHM2 models), resulting in increased susceptibility to CSD. Adapted with permission from *Journal of Headache and Pain* (100).

Overall, the findings suggest a model of CSD initiation in which a threshold depolarizing stimulus leads to Ca_V_-dependent glutamate release that overwhelms the reuptake capacity of the astrocytic glutamate transporters and activates synaptic and extrasynaptic NMDARs above the level necessary to generate a net self-sustaining inward current. This initiates the positive feedback cycle that ignites CSD by making the neuronal depolarization and the K^+^/glutamate elevations self-regenerative (5, 100, 118). This amplificatory cycle of glutamate/K^+^ release is potentiated in the FHM1 and FHM2 models as a consequence of either increased glutamate release or reduced rate of glutamate clearance. In both cases, the CSD-threshold levels of glutamate and NMDAR activation are reached with stimuli of lower intensity and more rapidly, resulting in increased susceptibility to CSD (Figure 3). Notably, increased glutamate release can also account for CSD facilitation in a genetic mouse model of nonhemiplegic MA (119).

A different mechanism may underlie facilitation of CSD in FHM3 mice. The facilitation of CSD in heterozygous *Scna1a^L1649Q^* mice was rescued by preferential inhibition of the persistent Na^+^ current with GS967 (88). It has been suggested that the larger accumulation of extracellular K^+^ at each action potential, due to increased persistent Na_V_1.1 current in interneurons, and the consequent build-up of [K^+^]_e_ and increased firing of excitatory neurons may underly the enhanced susceptibility to CSD in FHM3 mice (86, 88, 100, 120). Given the reciprocal relationship between K^+^ and glutamate, whereby an increased [K^+^]_e_ leads to increased glutamate release and decreased efficiency of glutamate reuptake, the proposed explanation for facilitation of CSD in FHM3 mice might be consistent with the model of CSD initiation and facilitation described above (100). In addition, it was proposed that an increased persistent Na_V_1.1 current in cortical pyramidal cells (121) might contribute to CSD facilitation in FHM3 (100).

Although these studies in FHM mouse models give insights into the key players likely involved in initiation of spontaneous CSDs in the brain of patients with HM (and possibly migraine), the mechanisms whereby specific triggering factors lead to CSD eruption in the brain of migraineurs remain largely unknown. Given the differential effect of FHM1 mutations on cortical excitatory and inhibitory neurotransmission and on synaptic plasticity at different synapses (57, 61, 109–111), it was proposed that the key pathogenic mechanism in HM (and possibly migraine) may be dysfunctional regulation of the excitatory-inhibitory (E/I) balance in specific neuronal circuits (57, 122). Considering that CSD is most easily induced in cortical upper dendritic layers (123–125), to favor CSD ignition, such dysfunction should probably lead to hyperactive excitatory synapses in upper layers and disinhibition of apical dendrites (perhaps with some metabolic compromise) (100). Whether this occurs, in which conditions and which neural circuits are involved, remain unknown.

*Cacna1a^R192Q^* mice show evidence of dysfunctional regulation of the E/I balance in specific cortical microcircuits, which are essential to prevent overexcitation (110, 111). Unexpectedly, the E/I balance was not skewed toward excitation, indeed during repetitive thalamic stimulation it was skewed toward inhibition. This is due to enhanced excitatory transmission at the synapses onto inhibitory interneurons and consequent enhancement of disynaptic feedforward and feedback inhibition, which may or may not counterbalance the increased recurrent excitation, depending on conditions (110, 111) (Figure 1). The responses to visual stimulation in awake *Cacna1a^R192Q^* mice were also consistent with a shift of the E/I balance toward inhibition (126). While the circuit alterations revealed by these and other studies in FHM1 mice (99, 127) may lead to alterations in sensory processing, it is difficult to envision how they may favor initiation of spontaneous CSDs (100). At any rate, considering the FHM1 cortex as hyperexcitable might be an oversimplification.

### Migraine-relevant pain behavior.

Some of the FHM mouse models exhibit migraine-relevant pain behavior. When subjected to novelty or restraint stress, homozygous *Cacna1a^R192Q^* mice showed behavioral changes suggestive of unilateral head pain, which were more frequent in females and normalized after administration of the antimigraine drug rizatriptan (128, 129). Some of these behaviors were more frequent and severe in *Cacna1a^S218L^* mice (129). Optogenetic induction of CSDs in freely behaving mice evoked a pain mimic that was longer lasting in heterozygous *Cacna1a^S218L^* mice than in WT mice (130). *Atp1a2^W887R^* mice showed enhanced cranial pain responses to systemic administration of the nitric oxide donor nitroglycerin (NTG) (114), which is known to induce a delayed migraine-like headache in migraineurs (131–133). In mice, NTG produces thermal and mechanical allodynia, responsive to the antimigraine drug sumatriptan (134). In *Atp1a2^W887R^* mice, orofacial mechanical hypersensitivity was elicited by low doses of NTG that were ineffective in WT mice (114).

**Figure 4:**
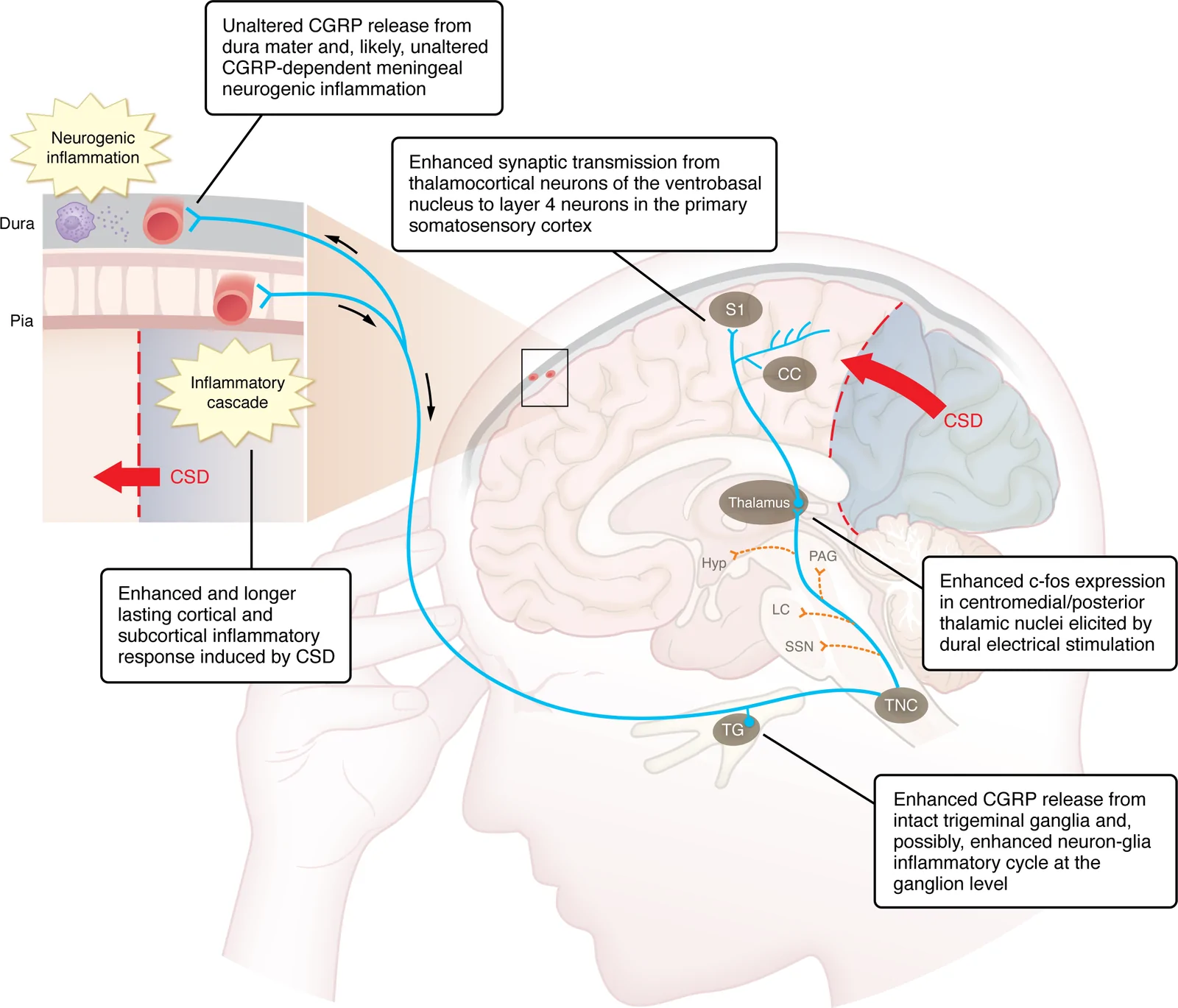
Functional alterations in the trigeminovascular pain pathway in FHM1 knockin mouse models. In FHM1 mice, a number of alterations in the trigeminovascular system might contribute to migraine-relevant pain behavior, including enhanced and longer lasting cortical and subcortical inflammatory responses induced by CSD; enhanced CGRP release from trigeminal ganglia and, possibly, enhanced neuron-glia inflammatory cycle at the ganglion level; enhanced c-fos expression in centromedial/posterior thalamic nuclei elicited by dural stimulation and enhanced synaptic transmission from thalamocortical neurons of the ventrobasal nucleus to layer 4 neurons in the primary somatosensory cortex. In contrast, migraine-relevant pain behavior is likely unrelated to CGRP-dependent meningeal neurogenic inflammation.

Given the key role of CGRP in the trigeminovascular pain pathway, CGRP release has been analyzed in *Cacna1a^R192Q^* mice. Neither basal nor K^+^-evoked CGRP release from dura mater were altered in adult homozygous *Cacna1a^R192Q^* mice (58, 135). This is likely consistent with the unaltered Ca_V_2.1 current in small capsaicin-sensitive *Cacna1a^R192Q^* TG neurons, which constitute the majority of small dural afferents (58), and with the lower fraction of CGRP-expressing neurons in TG of *Cacna1a^R192Q^* mice (136). Dural artery vasodilation induced in vivo by either endogenous (evoked by capsaicin) or exogenous CGRP was decreased in *Cacna1a^R192Q^* mice (135), suggesting unaltered CGRP release from trigeminal afferents together with downregulation and/or desensitization of blood vessel CGRP receptors. Thus, it is unlikely that enhanced CGRP-dependent meningeal neurogenic inflammation (8) contributes to the migraine-relevant pain phenotypes in FHM1 mice (Figure 4). K^+^-evoked (but not basal) CGRP release from intact trigeminal ganglia was increased in adult *Cacna1a^R192Q^* mice, consistent with the enhanced Ca_V_2.1 current and prolonged duration of action potentials in a subpopulation of TG neurons not innervating the dura (58). Basal CGRP release was increased in cultured TG neurons from *Cacna1a^R192Q^* pups (137), and multiple studies on these cultures (reviewed in refs. 138, 139) led to the suggestion that, in the ganglion, CGRP may function in a paracrine manner to stimulate satellite glial cells and neurons, which, by releasing signals like NO and cytokines, may further stimulate CGRP release. This would promote a positive feedback neuron-glia inflammatory cycle that may mediate sensitization of TG neurons, and this inflammatory cycle may be amplified in *Cacna1a^R192Q^* mice (Figure 4). Further work is necessary to establish whether enhanced release of CGRP (and other algogenic mediators) in the adult FHM1 TG leads to enhanced and prolonged peripheral trigeminal sensitization and contributes to migraine-relevant pain phenotypes.

Synaptic transmission from thalamocortical neurons of the ventrobasal nucleus (which comprises the primary thalamus sensory relay nucleus of trigeminovascular activation, ref. 4) to layer 4 neurons in the primary somatosensory cortex was enhanced in *Cacna1a^R192Q^* mice (110), as was c-fos expression elicited by dural electrical stimulation in centromedian/posterior thalamic nuclei (140) (Figure 4). It remains to be established whether these alterations contribute to migraine-relevant pain behavior in FHM1 mice.

CSD induced an enhanced and longer lasting cortical and subcortical inflammatory response in heterozygous *Cacna1a^S218L^* mice compared with that in WT mice, which was reduced by local block of NMDARs (130, 141) (Figure 4). This might contribute to the longer lasting CSD-triggered pain phenotype in FHM1 mice (130).

The cingulate cortex is activated during both spontaneous migraine attacks and during the premonitory phase of the migraine-like headache induced by NTG (132, 142). Some of the downstream regions to which the cingulate cortex is connected are involved in descending modulatory pathways implicated in central sensitization of trigeminovascular neurons in the trigeminal nucleus caudalis (TNC) (2, 3, 143). Sensitization of TNC neurons receiving convergent input from the meningeal nociceptors and facial skin underlies migraine cephalic allodynia (2). In *Atp1a2^W887R^* mice, restoring α2 NKA expression in the cingulate cortex (via local viral delivery of the *Atp1a2* gene) not only reversed the defective K^+^ and glutamate clearance and the increased output firing in layer 5 pyramidal cells but also strongly reduced NTG-induced orofacial mechanical hypersensitivity (114). This shows that altered neural activity in the cingulate cortex contributes to the enhanced cranial pain responses to NTG treatment in *Atp1a2^W887R^* mice.

## Other genes implicated in HM

A few studies have investigated the functional consequences of mutations in some of the other genes implicated in HM.

### PRRT2.

PRRT2 is a CNS neuron-specific membrane protein that mainly localizes in axons and concentrates at synaptic contacts (144). At the axonal level, PRRT2 interacts with the sodium channels Na_V_1.2 and Na_V_1.6, acting as a negative modulator of their membrane expression and function (145–147). This provides a molecular basis for the increase in intrinsic excitability in both excitatory (including cortical) neurons from *Prrt2*-knockout mice and iPSC-derived neurons from patients homozygous for the loss-of-function c.649dupC mutation (145–148), as well as for the heightened spontaneous network activity in cortical cultures from knockout mice (145, 148). At synaptic terminals, PRRT2 interacts with key components of the neurotransmitter release machinery and the Ca_V_2.1 channels (149–152), acting as a positive modulator of membrane expression of Ca_V_2.1 channels at the active zones (152). These synaptic interactions provide a molecular basis for the reduced hippocampal excitatory synaptic transmission and reduced probability of glutamate release when PRRT2 was either acutely silenced or constitutively inactivated (148, 150, 152). Conflicting findings were reported for inhibitory hippocampal synaptic transmission (148, 150). The increased intrinsic excitability of excitatory neurons appears as the key pathogenetic mechanism in the paroxysmal disorders caused by loss-of-function mutations in *PRRT2* (32) and in the behavioral phenotypes of *Prrt2*-knockout mice (146, 151, 153).

### SCN2A.

*SCN2A* encodes the pore-forming subunit of the neuronal voltage-gated sodium channel Na_V_1.2, which is predominantly expressed in brain excitatory neurons. Subcellular location and function change during development: Na_V_1.2 channels regulate axonal excitability during early postnatal life and action potential backpropagation, dendritic excitability, and synaptic function in mature pyramidal neurons (24). The HM-associated mutations affect the biophysical properties of recombinant human Na_V_1.2 channels, possibly with a net gain-of-function effect (44).

### SCL1A3.

*SCL1A3* encodes the glial glutamate transporter EAAT1 (GLAST in rodents), which is the main astrocytic glutamate transporter in the cerebellum but is also highly expressed in the cerebral cortex, where it participates together with GLT-1 in glutamate clearance by astrocytes during synaptic activity, although with a relatively minor role in many cortical areas (154–158). The mutation T387P, identified in a patient with HM, produces a loss of function of human recombinant EAAT1 (39), and a different mutation in another patient truncates the protein (41). Notably, CSD induction was not altered in GLAST-knockout mice, and it was facilitated in mice with 70% reduced expression of GLT-1 (159).

### SLC4A4.

*SLC4A4* encodes the electrogenic Na^+^-HCO3^–^ cotransporter NBCe1, whose splice variants are differentially expressed in various organs, including the brain (mainly in astrocytes) (160). Homozygous loss-of-function mutations in *SCL4A4* cause proximal renal tubular acidosis (161), which is associated with migraine (including HM) in a minority of patients (40, 42). The mutations associated with migraine led to cytosolic retention and near-total loss of transport activity of recombinant NBCe1, while the mutations not associated with migraine led to approximately 40% reduced transport activity (40). One may predict that loss of function of NBCe1 would lead to impaired glial acid secretion consequent to astrocytic depolarization during neuronal activity, and this would enhance the activity of NMDARs (which is steeply dependent on pH) (40, 162).

It remains unknown whether loss of function of NBCe1 or PRRT2 or gain of function of Na_V_1.2 affect CSD. Also unknown is whether these alterations or loss of function of GLAST affect the trigeminovascular system.

## Translational implications for patient therapy

The current treatment strategies for HM are empirical, based on single reports or small studies (with low level evidence), and suggest prophylactic treatments with verapamil, acetazolamide, flunarizine, lamotrigine (the latter only for aura; the first two also for abortive treatment) (refs. 15, 163 and references therein), and, more recently, with anti-CGRP monoclonal antibodies (ref. 164 and references therein). There is strong need for novel effective and safe therapies. Given that in HM long-lasting, severe aura symptoms may be more troublesome than the headache, a medication that can counteract the facilitated CSD initiation and propagation is in particular demand. The pathogenic mechanisms of CSD facilitation uncovered in FHM1 and FHM2 mouse models suggest that targeting the excessive glutamatergic neurotransmission and/or the excessive activation of NMDARs might be successful preventive therapeutic approaches. Given the role of CSD in triggering the headache mechanisms (6, 9, 10) and the role of glutamate and NMDARs in transmission of nociceptive information in the trigeminovascular pain pathway and in central sensitization (165–167), these approaches would probably also inhibit migraine pain. However, the vital roles of glutamate and NMDARs in normal brain function complicate therapeutic development. For example, despite evidence that intranasal application of ketamine (a relatively low-affinity NMDAR open channel blocker) reduces the severity and possibly duration of the aura symptoms in patients with HM (168, 169), and intravenous ketamine infusion relieves pain in retrospective case series of patients with refractory chronic migraine (170, 171), the clinical application of ketamine is limited by its propensity to cause (dose-dependent) psychotomimetic (and other adverse) symptoms (172).

Drugs that preferentially reduce overactivation of NMDARs consequent to excessive glutamatergic transmission while relatively sparing physiological NMDAR activity might be safe and potentially effective therapeutic options for patients with HM. Uncompetitive, fast off-rate blockers, like memantine, and negative allosteric modulators specifically targeting receptors containing the GluN2B subunit (GluN2B NAMs) are two drug classes with these characteristics. Memantine did not produce adverse effects in several large clinical trials showing its effectiveness for moderate-to-severe Alzheimer’s disease (173, 174), and phase I/II trials showed a good safety, tolerability profile for GluN2B NAMs (at sufficiently low doses) (175–181).

At low concentrations (≤10 μM) memantine affects less physiological synaptic transmission compared with ketamine and relatively more extrasynaptic than synaptic NMDARs (174, 182–186). The inhibition of NMDARs is strongly frequency dependent (187). Thus, memantine and its improved derivatives (NitroMemantines) should preferentially reduce overactivation of NMDARs in conditions of excessive glutamatergic transmission (186, 188, 189).

In in vivo preclinical studies, memantine injection (3–10 mg/kg) reversed the increased female-specific impulsive behavior in heterozygous *Atp1a2^G301R^* mice (73), prevented seizure-induced brainstem depolarizations and mortality in homozygous *Cacna1a*^S218L^ mice (190), and reduced the number of CSDs elicited by topical KCl in rats (191). Notably, there is uncertainty regarding the effective concentrations of memantine at the neuronal NMDARs in preclinical studies compared with those in the human brain at the 10–20 mg daily therapeutic dose (ranging between ≤1 and 10 μM, depending on the study, although the lower limit is probably more accurate) (174, 182, 192).

Interestingly, HM and seizure frequency were reduced by memantine treatment in a woman with very severe FHM2 (193), and there are case reports of patients with severe developmental and epileptic encephalopathy associated with *ATP1A2* mutations in whom memantine reduced the severe symptoms (194–197). Moreover, in small double-blind placebo-controlled and small open-label studies, memantine reduced the frequency and severity of migraine attacks in patients with migraine without aura as well as the frequency of aura in a retrospective case-series study, including patients with MA (198–203).

Two features of GluN2B NAMs make them interesting potential therapeutic options for HM: they exhibit activity-dependence and positive cooperativity between glutamate and GluN2B NAM binding (204, 205). Thus, they should be particularly effective at inhibiting NMDARs in conditions of excessive glutamatergic transmission. Supporting this, a GluN2B NAM (at a concentration that almost exclusively inhibits diheteromeric GluN2B receptors) rescued both the increased and prolonged activation of NMDARs and the facilitation of CSD initiation in cortical slices from *Atp1a2*^W887R^ mice, without affecting the activation of NMDARs and the CSD threshold in WT slices (115). Previous studies in rats in vivo had shown reduced susceptibility to experimental CSD by systemic administration of GluN2B NAMs (191, 206, 207). In a rat model of chronic migraine, increased tyrosine phosphorylation of GluN2B NMDARs, linked to excessive glutamatergic transmission, was shown to have a critical role in central sensitization and mechanical allodynia (167, 208). Thus, GluN2B NAMs might be able to ameliorate both aura and headache symptoms.

Although late-stage clinical development of first-generation GluN2B NAMs was prevented by narrow therapeutic windows, dose-limiting cardiovascular or CNS side effects and limited clinical efficacy, promising next-generation GluN2B NAMs are actively being developed for a variety of indications (175, 209). Some of these might be of particular potential interest for HM, including radiprodil (210, 211) or NP10679 (179).

Another class of allosteric drugs under development enhance the activity of EAAT2/GLT-1 glutamate transporters (EAAT2 PAMs, targeting excessive glutamate rather than excessive NMDAR activation) (212, 213). These include a recently described compound with robust antiseizure properties, excellent in vivo/in vitro safety profile, and satisfying pharmacokinetics (214). Although EAAT2 PAMs may offer clinical advantages over drugs, such as ceftriaxone, that increase EAAT2 expression (212, 215, 216), it remains to be established whether this therapeutic strategy, which affects glutamatergic neurotransmission throughout the brain, will be effective clinically without adverse effects (e.g., on learning/memory) (217). Of relevance for FHM2, ceftriaxone treatment only slightly increased the CSD threshold in *Atp1a2^W887R^* mice, because, in contrast with its enhancement of GLT-1 membrane expression in WT mice, it actually did not alter the density of GLT-1 glutamate transporters in perisynaptic astrocytic processes in *Atp1a2^W887R^* mice (64). It remains to be seen whether GLT-1 PAMs would be able to rescue the CSD facilitation in the FHM mutants.

The rescue of CSD facilitation in *Scna1a^L1649Q^* mice by GS967 (88) suggests that Na_V_ channel inhibitors that preferentially bind to the channel in the inactivated state might be safe and effective therapeutic options for patients with HM with gain of function of Na_V_ channels. Indeed, some case reports show efficacy of one such inhibitor, carbamazepine, in patients with FHM3/ERDB (23) and patients with HM with *PRRT2* mutations (218).

## Conclusions

The generation and functional characterization of several genetic mouse models of FHM, which all share increased susceptibility to experimental CSD, revealed that excessive cortical excitatory neurotransmission and overactivation of NMDARs underlie the facilitation of CSD in FHM1 and FHM2; excessive build-up of [K^+^]_e_ possibly underlies facilitation of CSD in FHM3. Thus, drugs that preferentially target overactivation of NMDARs while relatively sparing physiological activity might be safe and potentially effective therapeutic options for many patients with HM. Findings in FHM mice suggest that dysregulation of E/I balance in specific neural circuits may be the key pathogenic mechanism in HM. It is expected that future advances in understanding the cellular and circuit mechanisms underlying E/I balance dysregulation and its modulation by migraine triggers and brain states in FHM mouse models will give insights into the unknown mechanisms driving the onset of HM attacks and reveal novel therapeutic targets. New targeted therapies may also be revealed by future advances in understanding the mechanisms underlying migraine-relevant pain in FHM mice.

## Conflict of interest

The author has declared that no conflict of interest exists.

## Figures and Tables

**Table 3:**
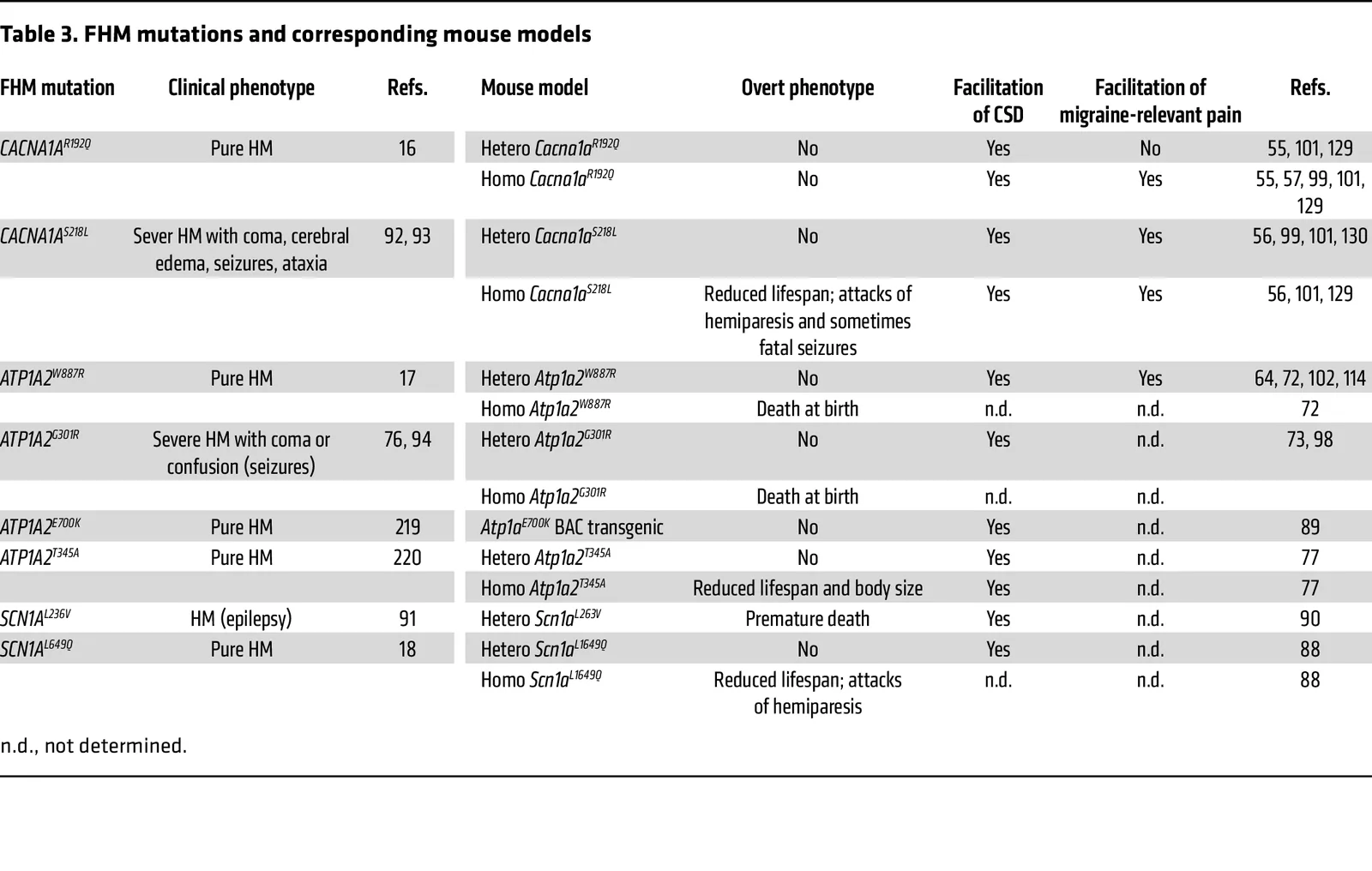
FHM mutations and corresponding mouse models

**Table 2:**
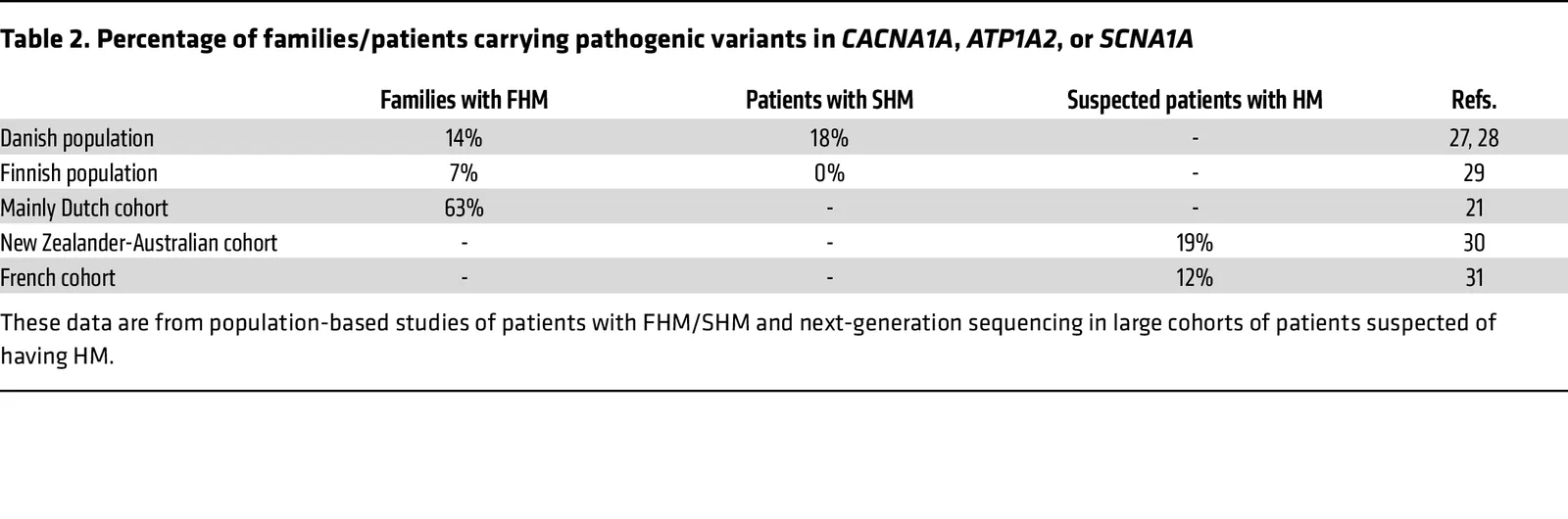
Percentage of families/patients carrying pathogenic variants in *CACNA1A*, *ATP1A2*, or *SCNA1A*

**Table 1:**
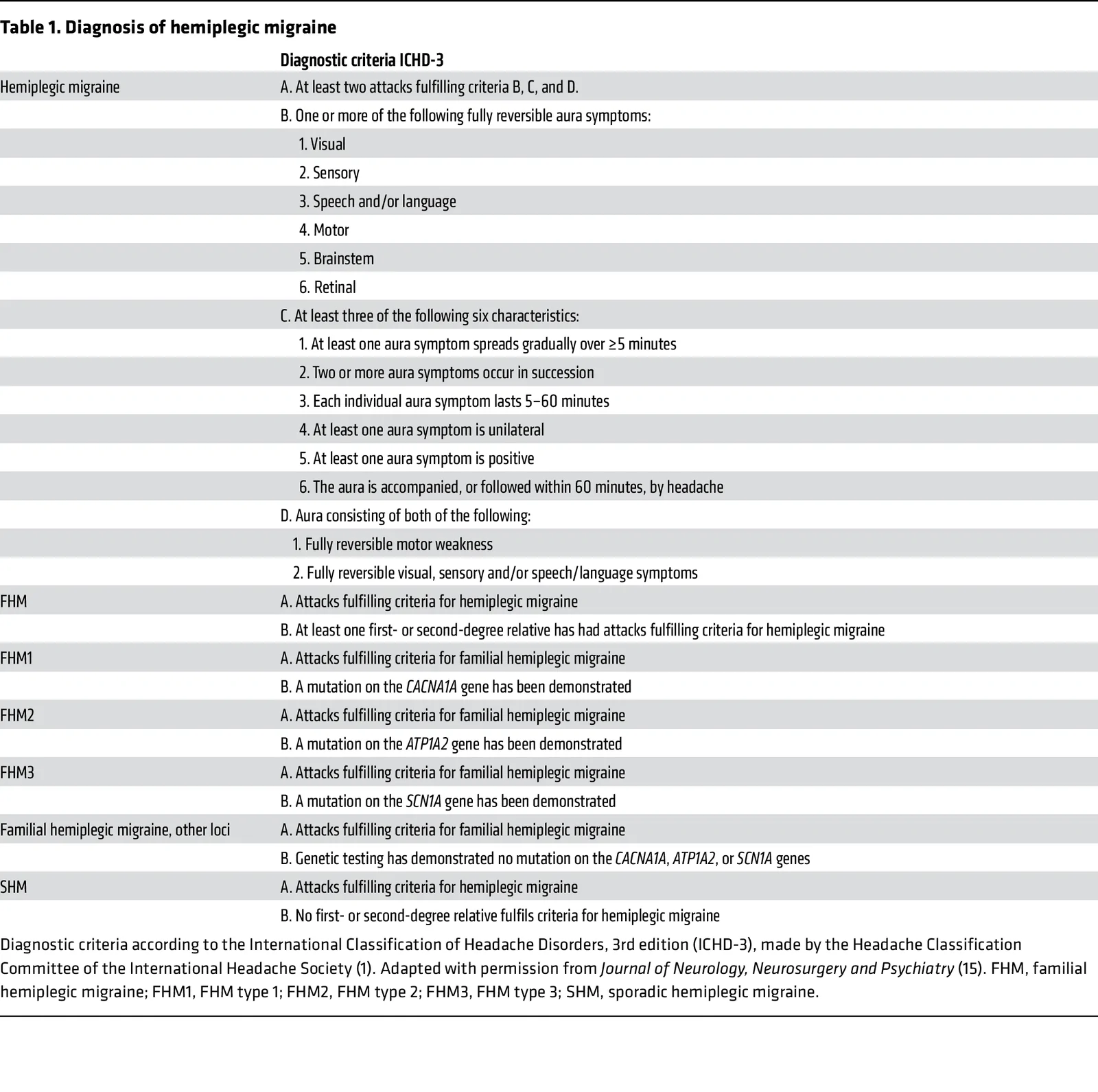
Diagnosis of hemiplegic migraine

## Version 1 09/15/2026

Electronic publication
